# Dynamic brain ADC variations over the cardiac cycle and their relation to tissue strain assessed with DENSE at high‐field MRI

**DOI:** 10.1002/mrm.29209

**Published:** 2022-03-28

**Authors:** Jacob‐Jan Sloots, Martijn Froeling, Geert Jan Biessels, Jaco J. M. Zwanenburg

**Affiliations:** ^1^ Center for Image Sciences University Medical Center Utrecht Utrecht The Netherlands; ^2^ Department of Neurology, UMC Utrecht Brain Center University Medical Center Utrecht Utrecht The Netherlands

**Keywords:** apparent diffusion coefficient (ADC), clearance, glymphatic, brain pulsations, human physiology

## Abstract

**Purpose:**

The ADC of brain tissue slightly varies over the cardiac cycle. This variation could reflect physiology, including mixing of the interstitial fluid, relevant for brain waste clearance. However, it is known from cardiac diffusion imaging that tissue deformation by itself affects the magnitude of the MRI signal, leading to artificial ADC variations as well. This study investigates to what extent tissue deformation causes artificial ADC variations in the brain.

**Theory and Methods:**

We implemented a high‐field MRI sequence with stimulated echo acquisition mode that simultaneously measures brain tissue deformation and ADC. Based on the measured tissue deformation, we simulated the artificial ADC variation by combining established theoretical frameworks and compared the results with the measured ADC variation. We acquired data in 8 healthy volunteers with diffusion weighting b = 300 and b = 1000 s/mm^2^.

**Results:**

Apparent diffusion coefficient variation was largest in the feet‐to‐head direction and showed the largest deviation from the mean ADC at peak systole. Artificial ADC variation estimated from tissue deformation was 1.3 ± 0.37·10^−5^ mm^2^/s in the feet‐to‐head direction for gray matter, and 0.75 ± 0.29·10^−5^ mm^2^/s for white matter. The measured ADC variation in the feet‐to‐head direction was 5.6·10^−5^ ± 1.5·10^−5^ mm^2^/s for gray matter and 3.2·10^−5^ ± 1.0·10^−5^ mm^2^/s for white matter, which was a factor of 3.5 ± 0.82 and 3.4 ± 0.57 larger than the artificial diffusion variations. The measured diffusion variations in the right‐to‐left/anterior‐to‐posterior direction were a factor of 1.5 ± 1.0/1.7 ± 1.4 and 2.0 ± 0.91/2.5 ± 0.94 larger than the artificial diffusion variations for gray matter and white matter, respectively.

**Conclusion:**

Apparent diffusion coefficient variations in the brain likely largely reflect physiology.

## 1 INTRODUCTION

The diffusion coefficient reflects the random motion of water molecules as induced by intrinsic thermal energy. It can be measured by applying a pulsed‐gradient MRI sequence in a certain direction, providing the ADC in the associated direction.^1^ This way, DWI provides a well‐defined diffusion measurement. The magnitude and anisotropy of multiple DWI measurements is used to study neuroanatomical microstructures in the human brain. Previous studies have found slight variations of ADC in brain tissue over the cardiac cycle.^2^, ^3^ Various physiological reasons have been proposed to explain these variations, including variation in amounts of intracellular and extracellular fluids and mixing or stirring of fluids in the interstitial space.^4^ As interstitial fluids are considered to be involved in the drainage of cerebral waste, dispersion effects like mixing or stirring might contribute to the clearance.^5^, ^6^

However, when the targeted tissue deforms during measurements, it has been demonstrated that the measured ADC can vary due to strain modulation of diffusion encoding.^7^, ^8^, ^9^ Contraction of the tissue increases the spatial frequency of the longitudinal modulation as imposed by the motion‐encoding gradient, while stretch causes a decrease in this frequency. These changes in spatial frequency change the effective b‐value experienced by the tissue.^9^ At the same time, tissue deformation leads to imperfect refocusing of the signal resulting in a lower magnitude.^10^ Especially in cardiac diffusion imaging, these effects have become most apparent because of the high tissue deformations in the beating in vivo heart.^7^ Thus, measured ADC variations can arise from “artificial” variations induced by tissue deformation that have no further physiological implication. It is yet unclear to what extent ADC variations in the brain can be explained by these “artificial” effects that tissue strains have on ADC measurements.

Strain correction of ADC variations require tissue strain maps. For cardiac diffusion imaging, strain maps are obtained from separately acquired data, typically using myocardial tagging, which induces dark “taglines” in the image from which tissue displacements and strains can be inferred.^11^, ^12^ As the analysis relies on visible taglines, these taglines need to be at least two voxels apart, which limits the motion sensitivity. For brain imaging, the sensitivity of such tagging sequences needs to be increased substantially. By encoding displacement information in the phase data rather than the magnitude data, arbitrary motion sensitivity can be obtained. Displacement encoding with stimulated echoes (DENSE) is an MRI tagging sequence that encodes tissue displacements in the MRI phase signal.^13^ At the same time, high motion sensitivity comes with high encoding gradients in the DENSE sequence, which induce considerable b‐values, and thus diffusion weighting in the magnitude images. Consequently, a DENSE sequence can simultaneously provide both strain data and diffusion data in the brain.

In this study, we investigate to what extent dynamic ADC variations over the cardiac cycle can be explained by artificial fluctuations in ADC induced by deformation of the tissue between the gradient pulses of the diffusion‐weighted sequence. To this end, we implemented a slice‐selective DENSE sequence that simultaneously provides data to measure brain‐tissue strain and ADC measurements over the cardiac cycle. We acquire data with b‐values of 300 and 1000 s/mm^2^, high enough to exclude perfusion effects from blood flow.^14^ Furthermore, we combine established theoretical frameworks on phase dispersion, tissue deformation, and their effects on the measured ADC.^9^, ^10^ We use the theory to simulate the artificial ADC variation based on measured tissue deformation and compare these artificial fluctuations in ADC to the measured ADC variation over the cardiac cycle. We show that tissue deformation indeed induces artificial ADC variations, but that these cannot fully account for the actual measured ADC variation, which leaves room for discussion about the physiological origin of the additional ADC variation.

## 2 THEORY

**FIGURE 1:**
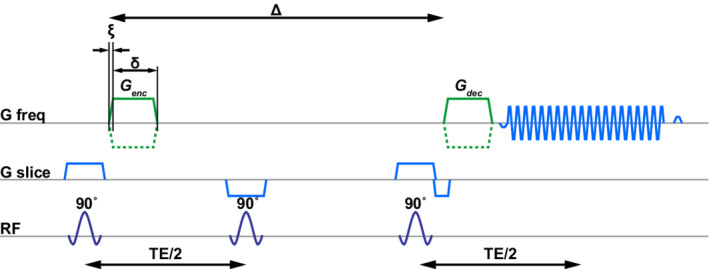
Schematic representation of the slice‐selective single‐shot displacement encoding with stimulated echoes (DENSE) sequence. The tissue's initial position is encoded in the longitudinal magnetization using G_enc_. After an evolution time Δ of 100 ms, the position of the tissue is decoded by applying G_dec_ (equal to G_enc_), which yields a signal phase that is proportional to the tissue displacement during Δ. At the same time, this scheme can be regarded as a STEAM diffusion sequence. The pulsed gradients induce diffusion weighting, which changes the signal magnitude. As a result, changing the gradient strength not only yields different displacement‐encoding sensitivity, but also a different diffusion weighting. For the brain, the unique situation arises where the pulsed gradients both meet the requirements for sufficient accuracy in the tissue‐motion field maps for strain computations, while at the same time reasonable diffusion weighting is achieved

Diffusion MRI encodes molecular diffusion effects in the NMR signal by using pulsed gradients (see Figure 1 for sequence design). However, tissue deformation that occurs during the time between these pulsed gradients leads to phase dispersion and a modified effective b‐value. These phenomena were already described by Wedeen et al^10^ and Reese et al,^9^ respectively. Under the assumption of a short gradient duration *δ* compared with the evolution time *Δ* after the second gradient lobe (see Figure 1), the stimulated echo signal M(r) in the presence of tissue deformation can be described as follows:

(1) $$\begin{array}{cc} M\left(r\right) & =\frac{M_{0}}{2}\cdot {∭}_{V}\exp\left(ir\cdot \left(k-k_{0}\right)\right)\mathrm{dV}\\ & \;\cdot \exp\left(-\Delta \cdot k^{T}\cdot D\cdot k\right), \end{array}$$

where Δ is the time between the pulsed gradients. The spatial frequency that results from the applied gradient pulses is represented by $k_{0}$. It changes to k as a result of tissue deformation, which we compute using the infinitesimal strain theory (see Supporting Information). In the remainder of this section, we revisit both phase dispersion and the effective b‐value separately, and eventually combine both factors to simulate the artificial ADC variation in the brain.

### 2.1 Phase dispersion

Incremental tissue deformation that accumulates during the time between the pulsed gradients leads to imperfect refocusing of the MRI signal. This effect is known as phase dispersion and results in a reduction of the magnitude of the MRI signal.^15^ The amount of phase dispersion not only depends on the incremental tissue deformation (represented by the deformation gradient tensor **F**), but also on the spatial frequency modulation (represented by $k_{0}$), leading to increased phase dispersion effects for a given tissue deformation as b‐values increase. The initial spatial frequency modulation $k_{0}$ of the longitudinal magnetization in a given voxel as induced by the pulsed gradients is given by $k_{0}=2\text{πγGδ}n$ (in mm^−1^, where n is the unit vector in the direction of encoding). The spatial frequency of the modulated magnetization changes as a result of the tissue deformation, yielding the following spatial frequency in the tissue just before the application of the second gradient pulse:

(2) $$k={\left(F^{-1}\right)}^{T}\cdot k_{0}.$$

If no deformation occurred in the time between the pulsed gradients, **F** would yield the identity matrix **I**.

Substituting r=xx+yy+zz and $\left(k-k_{0}\right)=\Delta_{k_{x}}x+\Delta_{k_{y}}y+\Delta_{k_{z}}z$ (**x**, **y**, and **z** unit vectors) in Eq. 1, we rewrite the volume integral by using the linearity property, as follows:

(3) $$\begin{array}{cc} & {∭}_{V}\exp\left(ir\cdot \left(k-k_{0}\right)\right)\mathrm{dV}\\ & ={∭}_{V}\exp\left(\mathrm{ix}\Delta k_{x}\right)\exp\left(\mathrm{iy}\Delta k_{y}\right)\exp\left(\mathrm{iz}\Delta k_{z}\right)\mathrm{dx}\;\mathrm{dy}\;\mathrm{dz}\\ & =\int \exp\left(\mathrm{ix}\Delta k_{x}\right)\mathrm{dx}\int \exp\left(\mathrm{iy}\Delta k_{y}\right)\mathrm{dy}\int \exp\left(\mathrm{iz}\Delta k_{z}\right)\mathrm{dz}. \end{array}$$

Each separate integral in Eq. 3 along the orthogonal directions *x*, *y*, and *z* can be solved analogous to the integral along *x*, as follows:

(4) $$\begin{array}{cc} \int_{-d_{x}/2}^{d_{x}/2}\exp\left(\mathrm{ix}\Delta k_{x}\right)\mathrm{dx} & ={\left[\frac{1}{i\Delta k_{x}}\exp\left(\mathrm{ix}\Delta k_{x}\right)\right]}_{-d_{x}/2}^{d_{x}/2}\\ & =\frac{2}{\Delta k_{x}}\left|\sin\left(\frac{d_{x}}{2}\Delta k_{x}\right)\right|, \end{array}$$

where *d*_*x*_ is the length of the voxel in the x‐direction.

From Eq. 2 it follows that for a given incremental tissue deformation, the difference in spatial frequency modulation $\left(k-k_{0}\right)$ will increase when $k_{0}$ increases. Consequently, larger $k_{0}$ leads to more signal attenuation due to enhanced phase dispersion effects, yielding an overestimated ADC when computed from two data sets with different spatial modulation frequencies, but equal tissue deformation.

### 2.2 Modified effective b‐value

Tissue deformation induces a different effective b‐value due to a modified spatial frequency. Under the assumption of a short gradient duration *δ* compared with the evolution time *Δ*, the factor in Eq. 1 related to diffusion can be rewritten in terms of the initial spatial frequency modulation $k_{0}$, as previously derived by Reese et al^9^:

(5) $$\exp\left(-\Delta \cdot k^{T}\cdot D\cdot k\right)=\exp\left(-\Delta \cdot {k_{0}}^{T}\cdot D^{\mathrm{obs}}\cdot k_{0}\right).$$

Ignoring the effect of the tissue deformation on the spatial modulation frequency of the longitudinal magnetization would lead to an observed diffusion coefficient **D** ^**obs**^ that is in general different from the actual diffusion coefficient **D**. The observed diffusion coefficient **D** ^**obs**^ is related to the actual diffusion coefficient **D** by

(6) $$D^{\mathrm{obs}}=\frac{1}{\Delta }\int_{0}^{\Delta }U^{-1}\left(t\right)\cdot D\cdot U^{-1}\left(t\right)\mathrm{dt},$$

where **U** is the stretch tensor (see Eq. S4 in the Supporting Information).^9^ From Eq. 6 it follows that the strain history of the tissue is encoded in **D** ^**obs**^. Even if the tissue deforms, but is again in its initial position during readout, **D** ^**obs**^ can differ from **D**. The observed diffusion coefficient reduces for positive strain (lower effective b‐values) and increases for negative strain (higher effective b‐values).

## 3 METHODS

### 3.1 Slice‐selective DENSE

The DENSE sequence consists of a motion encoding and decoding part, which analogous to velocity encoding, manipulate the phase of the MRI signal such that it becomes proportional to the displacement, relative to the point of encoding.^16^ Here we introduce a slice‐selective DENSE approach. The main difference compared with conventional DENSE is that the two encoding RF pulses are slice‐selective the same way as the third RF pulse (see Figure 1). This approach ensures that the TR and evolution time Δ are constant, and equal for each slice. At the same time, the slice‐selective approach requires RF pulses with high bandwidth (BW) to ensure proper slice profiles. The approach is therefore not well compatible with water selective excitation using low bandwidths for fat suppression, as used previously.^17^, ^18^ Instead, fat suppression is performed using the gradient reversal approach, in which the slice‐selective gradient of the second RF pulse is reversed (see Figure 1).^19^

Phase images acquired through DENSE can be transformed to tissue displacement maps, from which strain maps can be computed. At the same time, slice‐selective DENSE can be regarded as a STEAM diffusion sequence.^20^ The pulsed gradients induce a b‐value, leading to decreased signal in the magnitude images from which ADC maps can be derived.

### 3.2 Data acquisition

The study was approved by the institutional review board. Eight healthy volunteers (4 females, age 25 ± 4 years) were included, and written informed consent was obtained. The volunteers were scanned at 7 T (Philips Healthcare) using an 8‐channel transmit operating in quadrature mode and 32‐channel receive head coil (Nova Medical). In each subject, six slice‐selective DENSE series were acquired with only in‐plane motion encoding. To obtain motion encoding in the right‐to‐left (RL), anterior‐to‐posterior (AP), and feet‐to‐head (FH) direction, these series were acquired with different orientations: two sagittal series (in‐plane FH and AP encoding), two coronal series (in‐plane FH and RL encoding), and two transverse series (in‐plane AP and RL encoding). Each DENSE series consisted of 52 nontriggered repeated scans, half of the scans (26 repeated scans) with k_0_ = 55 mm^−1^ and the other half with k_0_ = 100 mm^−1^ (diffusion and motion‐encoding equivalent: b = 300/1000 s/mm^2^ and *D*_*enc*_ = 56/31 μm, respectively). The evolution time Δ was set to 100 ms to limit signal loss due to relaxation effects (predominantly determined by T_1_ in STEAM). Different k_0_ (ie, different b‐values) were obtained by varying the gradient strength G while keeping the effective gradient time parameters constant (δ = 9.5 ms, ξ = 0.4 ms). Each DENSE series provided two components of the deformation gradient tensor **J** by taking the in‐plane spatial derivatives from the motion‐encoded data (see Eq. 2 in the Supporting Information for the definition of **J**). Alternating encoding polarities were applied to distinguish between motion‐induced phase contributions and phase confounders. To ensure a fixed TR and constant diffusion effects with respect to Δ, the volumes were continuously acquired, resulting in displacement gradient maps randomly distributed over the cardiac cycle. As these displacements concern only the displacement developed during the mixing time Δ (100 ms in our case), we refer to these displacements and strains as *incremental* displacements and *incremental* strains. Physiological data from a pulse‐oximeter were simultaneously recorded to measure the cardiac interval position. Acquisition of a single DENSE series took 7 min regardless of heart rate. Further imaging parameters were as follows: 72 slices; resolution = 3 × 3 × 3 mm^3^; FOV = 240 × 240 × 216 mm^3^; SENSE = 2.6 (AP or RL, depending on acquisition orientation); single‐shot EPI (EPI factor = 35, EPI BW in the readout/phase encoding direction for sagittal and transverse orientation: 2.6 and 47 Hz/pixel; for coronal orientation: 3.5 and 56 Hz/pixel); B_1_ = 10 μT; TR = 7.5 s; and TE/2 = 24 ms.

A single‐shot multislice spin‐echo DTI data set was acquired for each volunteer, which was required to simulate ADC variations over the cardiac cycle based on incremental strain measurements (see Eq. 6). Diffusion tensor imaging was obtained with 16 directions, b‐values 300 and 1000 mm^2^/s, together with an additional b = 0 image. Different b‐values were obtained by varying the pulsed‐gradient strength G, while keeping time deltas constant (Δ = 36.4 ms, δ = 17 ms, ξ = 0.3 ms). The DTI data set was acquired with transverse orientation and acquired resolution of 2 × 2 × 2 mm^3^. Additional imaging parameters included FOV = 224 × 224 × 150 mm^3^; single‐shot EPI readout (EPI factor: 47; EPI BW in the readout/phase encoding direction: 2.5 and 35.9 Hz/pixel, respectively); SENSE factor = 2.4 (AP direction); TR = 8.4 s; and TE = 75 ms. The acquisition time was 5 min.

Two additional scans were acquired for data‐processing purposes. First, a T_1_‐weighted (T_1_w) turbo field echo scan was acquired as anatomical reference (acquired resolution = 1.00 × 1.00 × 1.00 mm^3^; FOV = 250 × 250 × 190 mm^3^; turbo field echo factor = 600; inversion delay = 1292 ms; SENSE = 2 (AP direction); flip angle = 5°; TR = 4.2 ms; TE = 1.97 ms; acquisition time = 2 min). Second, two B_0_ field maps were obtained: The first was acquired at the beginning of the scanning session to perform second‐order image‐based B_0_ shimming, and the second was acquired after shimming to allow for remaining geometric distortion corrections in the acquired DENSE images. A single B_0_ field map was reconstructed online from the phase difference of two successive gradient‐echo scans with fixed TR and different TE, as available from the vendor (acquired resolution = 3.50 × 3.50 × 3.50 mm^3^; FOV = 224 × 224 × 224 mm^3^; flip angle = 8°; TR = 3.9 ms; TE = 1.57 and 2.57 ms; scan duration = 25 s).

### 3.3 Postprocessing

**FIGURE 2:**
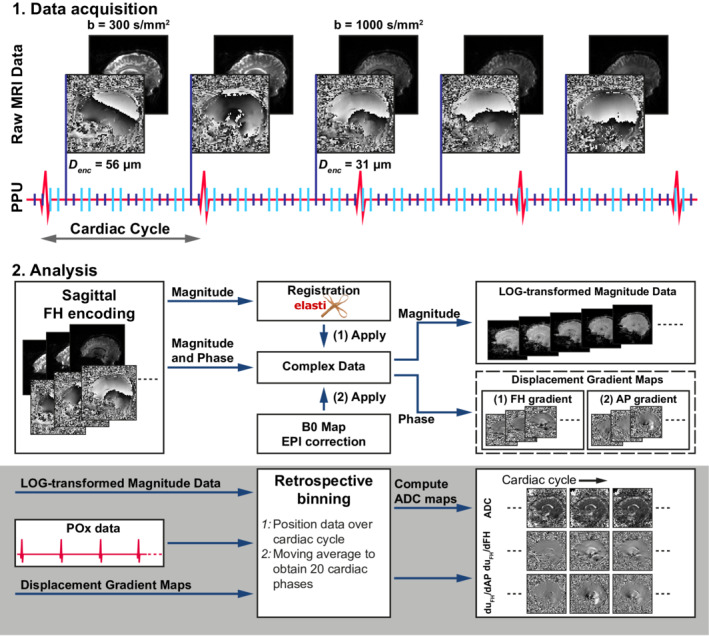
Schematic representation of DENSE data acquisition and analysis. (1) Slice‐selective single‐shot multislice DENSE images were acquired with two encoding sensitivities (b = 300 and b = 1000 s/mm^2^). Encoding (light blue) and decoding (dark blue) were interleaved for two slices, to speed up acquisition. (2) Magnitude data were used for registration, and the B0 map was used for EPI distortion correction. The derived parameters were applied to the complex data. From the complex data, magnitude and phase data were derived. The magnitude data were log‐transformed and the phase data were transformed into displacement maps, from which displacement gradient maps were computed. Log‐transformed data and incremental displacement gradient maps were repositioned over the cardiac cycle, and 20 binned maps over the cardiac cycle were constructed for the log‐transformed magnitude data and displacement gradient maps, using a moving average. Using the binned log‐transformed magnitude data, ADC maps were computed for 20 cardiac phases following Eq. 8. Abbreviations: AP, anterior‐to‐posterior; FH, feet‐to‐head; POx, pulse oximeter

Each acquired DENSE series was processed independently offline by using custom software written in *MATLAB* R2018b (The MathWorks, Natick, MA, USA). Per DENSE series, magnitude data were used for rigid registration of each repeated scan to the first repeated scan by using Elastix^21^ (see Figure 2 for analysis overview). Because adjacent slices were acquired at different positions in the cardiac cycle, only in‐plane degrees of freedom were used for registration. Subsequently, the shimmed B_0_ map was registered to the DENSE series and used for EPI distortion correction.^22^ Registration and distortion corrections were applied to the complex data using linear interpolation.

From the complex DENSE data, magnitude and phase data were obtained. Magnitude data (diffusion‐weighted) were log‐transformed. The phase data were transformed to displacement fields, from which two apparent displacement gradient maps were computed by taking in‐plane spatial derivatives. Unwrapping was performed under the assumption of small incremental displacement gradients ($\varepsilon_{\mathrm{xx}}\ll D_{\mathrm{enc}}/\Delta x$), where Δx is the voxel size.^17^ The limit of the incremental displacement gradients detectable over the evolution time Δ without phase wraps for b = 1000 was approximately 5·10^−2^, which is well over the expected incremental displacement gradient. At this point, the data still included static background contributions. Averaging the data from the opposite gradient polarities yielded this static background offset, which was subtracted from the data.

#### 3.3.1 Retrospective binning

For the DENSE data set, 20 cardiac phases of ADC and incremental displacement gradients were obtained. To this end, log‐transformed magnitude data and incremental displacement gradient maps were ordered over the cardiac cycle by using the position of acquisition that followed from the recorded physiological trace. Each DENSE data set provided 52 frames of displacement gradient maps (both k_0_ = 55 mm^−1^ and k_0_ = 100 mm^−1^ data) distributed over the cardiac cycle. The log‐magnitude data were processed separately as per diffusion weighting, resulting in 26 frames per b‐value. Subsequently, a moving average window with a width of 10% of the cardiac interval was applied to the data to generate 20 cardiac frames. Sometimes, no data were available in the bin. In those cases, the values of the two neighboring data points on either side of the bin were averaged. This happened in less than 5% of cases for the diffusion‐weighted data and in less than 1% of cases for displacement gradient maps.

#### 3.3.2 Incremental strain tensor maps

For each subject, all six slice‐selective DENSE series were combined by using a group‐wise rigid registration with Elastix and third‐order b‐spline interpolation.^21^ These series yielded the incremental displacement gradient maps, from which Cauchy's strain tensor was derived. The procedure to derive Cauchy's strain tensor from the DENSE series has been summarized in the Supporting Information.^23^ Cauchy's strain tensor is a linearization of the general Lagrangian strain tensor, for which infinitesimal strain is assumed, which is ‖∇u‖ ≪ 1. The observed displacement gradients for brain tissue are maximally 5·10^−3^ [4; 5], which justifies the use of Cauchy's strain tensor, from which the incremental volumetric strain [1] and shear strain [3] curves were computed. Furthermore, volumetric strain^24^ and shear strain^25^ curves were computed from Cauchy's strain tensor. To reduce the intersubject variability of strain curves due to noise in the measurements, these curves were overlaid between subjects over the cardiac cycle as follows. For each subject, the mean incremental volumetric strain over the cardiac cycle was subtracted, resulting in volumetric strain curves oscillating around 0. Furthermore, the minimum incremental shear strain value over the cardiac cycle was subtracted from the shear strain curve, so that shear strain starts and ends at zero.

#### 3.3.3 T_1_‐weighted data and diffusion tensor

For each subject, the T_1_‐weighted anatomical scan was registered to the DENSE data. The full diffusion tensor was reconstructed offline from the DTI data using Explore DTI.^26^ Only the DTI data associated with b = 300 and b = 1000 were used to obtain a representative comparison to the ADC measurements obtained through DENSE. To correct for EPI distortions, nonlinear b‐spline regularized registration with the registered T_1_‐weighted scan as reference was used.

### 3.4 Artificial ADC variations

Artificial ADC variations, induced by tissue deformations, were calculated based on the theoretical framework (see section 2.2). The acquired DTI data and measured incremental displacement gradient tensor from the DENSE data over the cardiac cycle were used to simulate these artificial variations. By combining both phase dispersion and effective b‐value contributions, the magnitude of the MRI signal is described as

(7) $$\begin{array}{cc} \left|M_{d}\right| & =\frac{M_{0}}{2}\cdot \frac{2}{\Delta k_{x}}\left|\sin\left(\frac{d_{x}}{2}\Delta k_{x}\right)\right|\cdot \frac{2}{\Delta k_{y}}\left|\sin\left(\frac{d_{y}}{2}\Delta k_{y}\right)\right|\\ & \;\cdot \frac{2}{\Delta k_{z}}\left|\sin\left(\frac{d_{z}}{2}\Delta k_{z}\right)\right|\cdot \exp\left(-bD_{\mathrm{obs}}\right). \end{array}$$

The complete linearization of the incremental strain tensor and its implementation in the *MATLAB* software are described in the Supporting Information.

In predicting the ADC variation over the cardiac cycle, it is important to note that the ADC prediction depends on the log transform of Eq. 7. Log‐transforming Eq. 7 will result in a summation rather than a product, from which the predicted ADC variation can be derived. Consequently, the ADC variation does not depend on M_0_. The artificial (deformation‐induced) component of the variations in ADC were computed voxel‐wise for each of the three diffusion directions available from the DENSE magnitude images: RL, AP, and FH.

### 3.5 Procedure for analysis of ADC variations

For both measured and artificial ADC variations, ADC maps for each cardiac phase (*cp*) were computed from the log‐magnitude data with diffusion weighting, using the following equation:

(8) $$\mathrm{ADC}\left(\mathrm{cp}\right)=\frac{M_{300}^{\ln}\left(\mathrm{cp}\right)-M_{1000}^{\ln}\left(\mathrm{cp}\right)}{1000-300}.$$

Here, $M_{300}^{\ln}$ and $M_{1000}^{\ln}$ are the log‐transformed magnitude data with diffusion weighting of b = 300 and b = 1000, respectively. The ADC deviation (dADC) from the mean ADC (ADC_mean_) over the cardiac cycle was obtained by using

(9) $$\text{dADC}\left(\mathrm{cp}\right)=\mathrm{ADC}\left(\mathrm{cp}\right)-{\mathrm{ADC}}_{\text{mean}}.$$

The time dependency (*cp*) of dADC will be omitted in further references for brevity.

For the intersubject analysis, ADC variation curves were synchronized. To this end, peak incremental shear strains per subject were positioned at 30% of the cardiac interval, and associated ADC curves were shifted accordingly. Furthermore, the maximum temporal ADC change over the cardiac cycle (ΔADC) was calculated for a region of interest (ROI) as follows:

(10) $$\text{ΔADC}={\mathrm{ADC}}_{\max}-{\mathrm{ADC}}_{\min}\;,$$

where ADC_max_ and ADC_min_ indicate the maximum and minimum ADC average in the ROI over the cardiac cycle, respectively. Two ROIs were defined: the white‐matter (WM) and gray‐matter (GM) region. Conservative masks were created to avoid partial volume effects from large vessels and CSF. The T_1_‐weighted data were first registered to the DENSE data and subsequently segmented using the Computational Anatomy Toolbox (*CAT12*, version 1615; Jena University Hospital, Departments of Psychiatry and Neurology) for Statistical Parametric Mapping (*SPM12*, version 7771; Wellcome Trust Center for Neuroimaging, University College London). The conservative ROI masks were obtained using a probability threshold of 90% for WM and GM, respectively. These masks were intersected by a mask in which all voxels with CSF probability larger than 0 had been disregarded, followed by one additional city‐block erosion step. Due to this stringent approach and low spatial resolution, the GM mask consisted practically of deep GM only.

## 4 RESULTS

### 4.1 Tissue deformation and ADC

**FIGURE 3:**
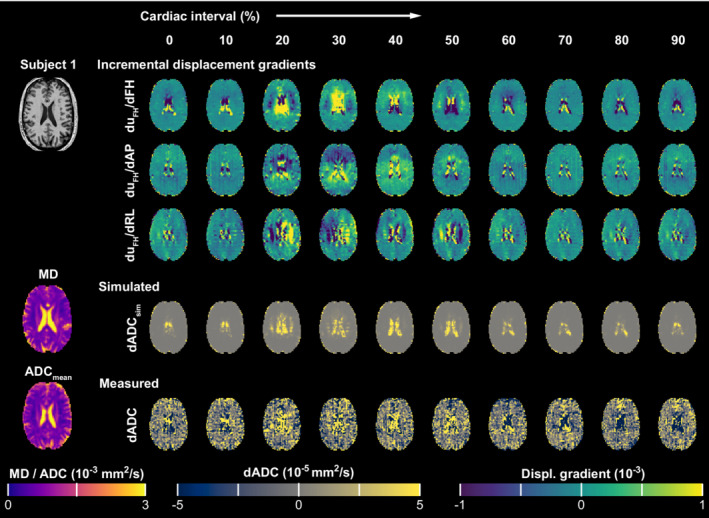
Apparent diffusion coefficient deviations from the mean (dADC) and incremental displacement gradient maps over the cardiac cycle for Subject 1. Cardiac phases are represented with peak incremental shear strain at 30% of the cardiac cycle. Although 20 cardiac phases were obtained, 10 are represented here. Slice‐selective DENSE data were acquired with FH encoding, which implies sagittal and coronal orientation. Here, we present the data in a transverse orientation. On the left, the T_1_‐weighted image is shown as an anatomical reference. Below it, the measured mean diffusion in the FH direction is represented by the reconstructed mean diffusivity (MD) and the mean ADC maps on the left, obtained through the DTI and DENSE data, respectively. On the right, the upper three rows represent the incremental displacement gradient fields observed over time Δ (100 ms). The duFH/dFH component was obtained with both sagittal and coronal orientation and averaged. The additional duFH/dAP and duFH/dRL components were obtained with sagittal and coronal acquisition orientation, respectively. The bottom two rows show the ADC deviation from the mean over the cardiac cycle, as simulated (dADCsim) and measured dADC, respectively. Abbreviation: RL, right‐to‐left

Average ADC values over the cardiac cycle obtained through DENSE compared well with the mean diffusivity (MD) values obtained with conventional DTI scanning. Overall, a slightly lower mean ADC (ADC_mean_) was found compared with MD obtained with DTI: 7.10 ± 0.38·10^−4^ mm^2^/s versus 8.15 ± 0.28·10^−4^ mm^2^/s. Figure 3 shows example maps for one of the subjects for data encoded in the FH direction. On the top left, T_1_‐weighted data are shown for anatomical reference. On the bottom left, MD and ADC maps are shown as obtained from the DTI and DENSE data, respectively. Because only in‐plane encoding was used, FH encoding implies that DENSE data were acquired with sagittal and coronal orientation.

Three incremental displacement gradient maps (first three rows) over the cardiac cycle shown were obtained through the phase data. The strain‐induced simulated artificial ADC deviations (dADC_sim_) and the actual measured deviations dADC over the cardiac cycle are represented in rows 4 and 5, respectively.

### 4.2 Simulated and measured ADC deviations

**FIGURE 4:**
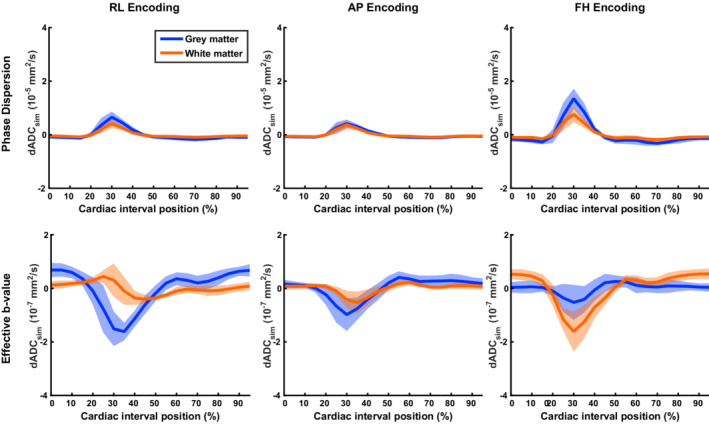
Simulated phase dispersion and effective b‐value contributions that constitute the dADCsim represented as the deviation from the MD over the cardiac cycle MD. Please note the difference in scaling along the y‐axis. The ADC variations were simulated voxel‐wise, and the results were averaged over the conservative masks, avoiding blood and CSF signals. From the DTI data, MD values along the associated direction are indicated per subject, in the legend. The blue and red solid lines represent the mean dADCsim over all subjects, whereas the shaded areas indicate the SD over subjects for white matter and gray matter, respectively. The largest variation was observed in the FH direction. Phase dispersion effects contributed to a dADCsim increase at peak systole, whereas the effective b‐value contributed to a slight decrease in simulated dADCsim at peak systole. Furthermore, phase‐dispersion effects were two orders of magnitude larger than the effective b‐value contribution. Because dADC is derived from the log‐transformed magnitude data, multiplications become summations and the overall simulated dADCsim is obtained by summing dADCsim for phase dispersion with dADCsim for the effective b‐value

Simulated ADC variations consist of two components: phase dispersion effects and modulated effective b‐value. Figure 4 represents the calculated artificial contribution of these components separately. The effect of phase dispersion on ADC variations is two orders of magnitude larger than the modulation of the effective b‐value. The phase dispersion is responsible for an increase in the diffusion coefficient, whereas the effective b‐value component results in a reduced diffusion coefficient at peak systole.

**FIGURE 5:**
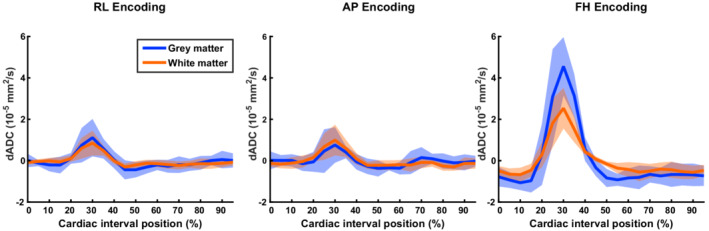
Measured dADC represented as the deviation from the mean ADC over the cardiac cycle for gray matter (blue) and white matter (red). Each orthogonal encoding direction was acquired 2 times, each with a different acquisition orientation for which the average is represented here (see Figures S1 and S2 for separate results per acquisition orientation and subject for GM and WM, respectively). Curves over the cardiac cycle were obtained by averaging over the conservative masks, avoiding blood and CSF signals. Curves between subjects were synchronized such that peak incremental shear strain occurred at 30% of the cardiac interval. The solid lines represent the mean dADC over all subjects, whereas the shaded area indicates the SD. The largest dADC was observed in the FH direction

**FIGURE 6:**
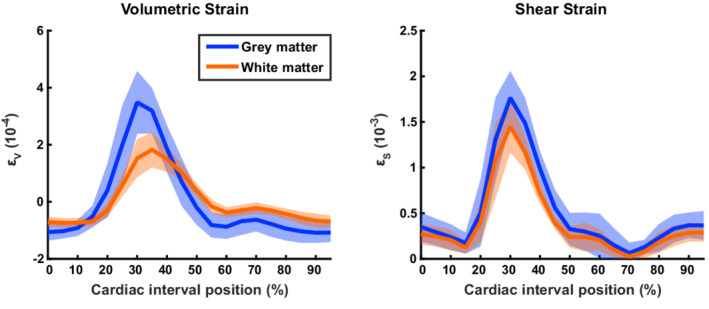
Incremental curves of volumetric strain and shear strain for gray matter (blue) and white matter (red). Peak incremental shear strains per subject were placed at 30% of the cardiac interval. Shifts required to obtain this result were applied to all associated curves (eg, volumetric strain and dADC). Furthermore, volumetric strain and shear strain were normalized over the cardiac cycle. To this end, the mean volumetric strain per subject was subtracted from the associated curve, so that volumetric strain curves oscillated around 0. For shear strain, the minimum incremental shear strain over the cardiac cycle was subtracted, resulting in shear strain curves touching to zero

Measured ADC values deviated from the mean ADC value over the cardiac cycle for each of the three investigated orthogonal directions. Figure 5 shows dADC for each encoding direction, where dADC with the same encoding direction but different acquisition orientation was averaged (see Figures S1 and S2 for dADC curves per acquisition orientation and per subject for GM and WM, respectively). For the analysis, peak incremental shear strains were synchronized, which resulted in a relative shift between subjects of at most two cardiac phases (which is equal to 10% of the cardiac interval). The largest dADC was observed at peak incremental shear strain (by definition at 30% of the cardiac interval) in the FH direction. The incremental shear strain curves computed from the incremental strain tensor are shown in Figure 6 together with the incremental volumetric strain. The maximum temporal ADC change (ΔADC) in the FH direction was 5.6·10^−5^ ± 1.5·10^−5^ mm^2^/s in GM (mean ± SD across subjects) and 3.2·10^−5^ ± 1.0·10^−5^ mm^2^/s in WM. Maximum temporal ADC change in the RL and AP direction were slightly lower: 1.6·10^−5^ ± 1.0·10^−5^ and 1.1·10^−5^ ± 0.94·10^−5^ mm^2^/s, respectively, in GM. In WM, these values were 1.2 ·10^−5^ ± 0.65·10^−5^ and 1.3·10^−5^ ± 0.76·10^−5^ mm^2^/s, respectively. Furthermore, intersubject variability in dADC at peak systole (observed primarily in the FH direction) was mostly explained by peak incremental shear strain rather than mean ADC: For the GM and WM combined, the coefficients of determination (R^2^) in the RL, AP, and FH direction for peak shear strain were 0.71 (*p* = 0.008), 0.69 (*p* = 0.011) and 0.60 (*p* = 0.023), whereas for mean ADC these coefficients were 0.06 (*p* = 0.55), 0.013 (*p* = 0.79) and 0.04 (*p* = 0.63), respectively.

### 4.3 Correlation tissue strain and measured ADC deviation

The ADC deviation for the GM and WM ROIs combined correlated best with tissue shear strain increments in all three orthogonal directions, indicating that shear strain was the best predictor of the amount of ADC variation measured. The overall coefficient of determination (R^2^) was calculated using the mean traces over the cardiac cycle for dADC, volumetric strain and shear strain, rather than averaging the individual coefficients of determination per subject. For both volumetric strain and shear strain, this coefficient of determination was highest in the FH direction (with GM and WM ROIs combined): 0.73 and 0.94, respectively. Correlation plots and coefficients of determination per subject are presented in Figures S3‐S5, for combined and separate GM and WM ROIs, respectively.

### 4.4 Corrected ADC deviation

**FIGURE 7:**
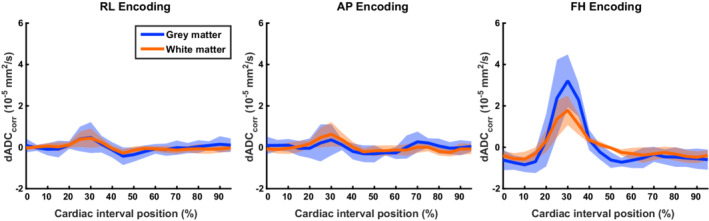
Corrected measured ADC deviations (dADC_corr_) over the cardiac cycle for gray matter (blue) and white matter (red). Results were obtained by subtracting simulated dADC induced by tissue deformation from measured dADC. Because the measured dADC deviations were on average 2.2 ± 1.1 (gray matter) and 2.6 ± 0.82 (white matter) times larger than simulated deviations, dADC_corr_ is still of the same order of magnitude as the measured dADCs (see Figure 5). These corrected ADC deviations from the mean ADC cannot be explained by intrinsic artificial signal variations arising from tissue deformation

Measured ADC deviations from the mean ADC were corrected for simulated ADC deviations by subtracting dADC_sim_ from the measured dADC. The simulated dADC resulted from the summation of both phase dispersion and effective b‐value contributions (separately shown in Figure 4). The corrected dADC (dADC_corr_) is presented in Figure 7. The ratio between dADC and dADC_sim_ in the GM was on average 1.5 ± 1.0, 1.7 ± 1.4 and 3.5 ± 0.82 in the RL, AP and FH direction, respectively. In the WM, the respective ratios were 2.0 ± 0.91, 2.5 ± 0.94, and 3.4 ± 0.57.

## 5 DISCUSSION

We developed a comprehensive method to investigate to what extent ADC variations over the cardiac cycle in the brain can be explained by artificial variations induced by tissue deformation. We combined principles of DENSE and DTI in a single MRI sequence, which could be regarded as either a slice‐selective DENSE sequence or a STEAM diffusion sequence. This way, strain and ADC measurements could be obtained simultaneously from the phase and magnitude data, respectively. We measured ADC variation over the cardiac cycle in 8 subjects, for three orthogonal directions (RL, AP, and FH) and compared the results with artificial ADC variations based on the measured tissue strain tensor.

The temporal profile of the artificial dADC showed good similarities with the measured dADC, although the amplitude of the simulated effects was much lower. These similarities were especially observed for the FH direction, because of the relatively high amplitudes in that direction. Phase dispersion effects dominated the overall artificial ADC variation, its effect being two orders of magnitude larger as compared with the effective b‐value contribution. We found systematically larger MDs than ADCs obtained through DTI reconstructions and DENSE, respectively. The use of these slightly larger MDs in the simulations only affected the effective b‐value contribution and resulted in a slight overestimation of this effect. However, because of the large difference between phase dispersion and effective b‐value contributions, the effect on the outcome of the artificial ADC variation was negligible. We also assumed a constant strain rate over the evolution time Δ, resulting in a piecewise continuous tissue deformation as a function of cardiac phase. This assumption only affected the effective b‐value contributions and therefore had only a minor impact on the overall result. In fact, it would have been sufficient to consider only the phase dispersion contribution and disregard the effective b‐value altogether. As for the discrepancy between MDs and ADCs, this is most likely explained by differences in the time between the pulsed gradients (ie, the diffusion time). The diffusion time was considerably longer for DENSE compared with DTI: 100 ms versus 36.4 ms, respectively. Mathematical models have shown that the effective diffusion coefficients decrease as evolution times increase.^27^

Measured ADC variation was up to 3 times larger than the artificial ADC variation, depending on the encoding direction. The largest ADC variation was observed in the FH direction, which is consistent with larger strains associated for that direction.^17^, ^18^ Measured ADC variations correlated best with incremental shear strains and showed a brief peak at peak systole. Intersubject differences at peak systole in measured dADC were also largely explained by differences in incremental shear strains rather than differences in mean ADC values. The calculated phase dispersion effects showed that in the analysis of diffusion parameters, it is important to take shear strains into account. These effects might also be relevant for simultaneous DTI and MR elastography measurements, in which additional shear strains are induced by an external actuator, as is commonly used in conventional MR elastography.^28^ Meanwhile, incremental volumetric strain correlated less with measured ADC variations. Volumetric strain curves were generally more stretched in time compared with incremental shear strain, which also indicates that volumetric strain variations are not the main cause of shear strain.

The measured ΔADC ranged between 1.1·10^−5^ ± 0.94·10^−5^ and 5.6·10^−5^ ± 1.5·10^−5^ mm^2^/s. Largest ΔADC was observed in the FH direction, which was still one to two orders of magnitude smaller than ΔADC reported in literature.^2^, ^29^, ^30^ Ohno et al and Osawa et al have reported large ΔADC of approximately 0.24·10^−3^ mm^2^/s. However, in these studies the ΔADC was calculated on a voxel‐by‐voxel basis rather than on an ROI basis.^29^, ^30^ Calculating ΔADC is sensitive to noise, as it involves a difference between minimal and maximal values, and will tend to result in an overestimation. By using an ROI‐based approach instead, the SNR will increase, yielding a ΔADC that is more reliable. Nakamura et al used this approach and found a ΔADC of 0.07·10^−3^ mm^2^/s. Although this finding is more in line with the results we obtained, the difference is still well over a factor of 3. The remaining difference is probably explained by the use of b = 0 data in the referenced studies.^2^ It is known from the intravoxel incoherent motion model that low b‐values reflect signal variations from the blood pool and perfusion.^31^ By using only b = 0 and b = 1000 data, obtained ADC variations will include blood flow pulsations reflected by an increased ADC at peak systole. Some studies specifically targeted the blood pool using the intravoxel incoherent motion model as a surrogate for microvascular pulsatility.^14^, ^32^ In this work, however, we used high enough b‐values (300 and 1000, equivalent to displacement encoding of 56 and 31 μm) to exclude any contributions from blood or perfusion in the reconstructed ADC signal, which did not allow for a further investigation of these effects. The findings reported by Federau et al, however, indicate that perfusion effects in the measured signal are already reduced to less than 2% at b = 300.^14^ Furthermore, because there is evidence that volumetric strain reflects blood volume pulsations,^18^, ^24^, ^33^ the lower correlation of the volumetric strain with measured ADC variations suggests that the measured ADC variations were not dominated by blood volume, although higher noise levels in the volumetric strain measurements could have reduced the observed correlation as well.^18^ In addition, it is a limitation of this study that the acquisition of a data set with two b‐values already took 7 min. Adding additional b‐values would therefore result in infeasibly prolonged protocols. More advanced encoding routines could potentially reduce the acquisition time. Furthermore, it must be acknowledged that we did not reproduce the ADC variation by using a Stejskal‐Tanner spin‐echo sequence as reported in literature. Therefore, we could not validate our method against a conventional DTI sequence.

In this work, ADC and strain measurements were binned retrospectively over the cardiac cycle. This approach ensured a fixed TR for all slices independent of heart rate, yet resulted in data points being distributed randomly across the cardiac cycle. Consequently, data density and, therefore, SNR, in both strain and ADC measurements may differ for different cardiac phases, which was reduced by the use of the moving average window. Prospective cardiac triggering facilitates more direct control of SNR distribution over the cardiac cycle, because it enables fixed time delays with respect to the cardiac R‐top.^17^, ^18^ While this approach is frequently used in literature to assess ADC variation over the cardiac cycle, it makes the TR heartbeat‐dependent. Especially when TRs are short (eg, two R‐R intervals^29^, ^30^), variations in the subject's heart rate lead to unwanted signal variations due to T_1_ relaxation. Federau et al noticed this dependency between heart rate and TR, and minimized the effect by applying a minimum TR of 5 s.^14^ However, T_1_s are generally longer at higher field strengths, and variations in TR just over 5 s still may yield signal variation. Moreover, we were also interested in fluid contributions from CSF and interstitial fluid, which have longer T_1_s compared with tissues like GM and WM. Although the TR in this work is still not 2 times the T_1_ of CSF,^34^ it does not vary between measurements. In addition, we have not assessed ADC variations due to respiration, as our previous study showed that brain‐tissue deformations are driven primarily by the cardiac cycle, with respiration‐induced strains being a factor of 5 smaller for a full inspiration.^17^ These respiration‐induced strains will be even smaller for shorter mixing times (100 ms for the current study).

The ratio between artificial and measured ADC variations implies a possible physiological component that can explain the difference. In particular, the strong correlation of measured ADC variations with incremental shear strain indicates an underlying effect responsible for increased ADC variation. Although the findings in this work are intended to provoke further research in the underlying mechanisms, we think that mixing or stirring of the interstitial fluid or fluids in perivascular spaces driven by tissue deformation contributes to the observed variation in ADC.

## 6 CONCLUSIONS

The developed slice‐selective DENSE sequence is capable of simultaneously measuring ADC and strain variations of brain tissue. By combining these results with a single DTI data set, we were able to successfully estimate the artificial ADC variations induced by tissue deformation. Measured ADC variations were up to 3 times larger than the artificial variations, which is probably explained by a physiological effect. Here, future research is welcome to propose physiological effects that are responsible for the observed differences. We hypothesize that mixing or stirring of the interstitial fluid or perivascular spaces is driven by tissue deformation. Although further investigation is required to substantiate this hypothesis, it is conceivable that this additional mixing adds to an increased ADC variation over the cardiac cycle. This method provides a tool to study this effect and holds the potential to serve as a way to detect abnormalities in ADC variations in disease.

## FUNDING INFORMATION

The Netherlands Organization for Scientific Research (Vici Grant 918.16.616) and the European Union's Horizon 2020 Research and Innovation Program (666881)

## Supporting information


**Figure S1.** Measured ADC deviation (dADC) curves over the cardiac cycle separately shown per acquisition orientation and encoding direction. Curves over the cardiac cycle were obtained by averaging over the conservative gray‐matter (GM) mask, avoiding blood and CSF signals. Mean ADC values over the cardiac cycle per subject, obtained for the associated acquisition orientation and encoding direction are indicated in the legend. Curves between subjects were synchronized such that peak incremental shear strain occurred at 30% of the cardiac interval. The dotted black line represents the mean dADC over all subjects, whereas the gray shaded area indicates the SD. The largest dADC was observed in the feet–head direction
**Figure S2.** Measured dADC curves over the cardiac cycle separately shown per acquisition orientation and encoding direction. Curves over the cardiac cycle were obtained by averaging over the conservative white‐matter (WM) mask, avoiding blood and CSF signals. Mean ADC values over the cardiac cycle per subject, obtained for the associated acquisition orientation and encoding direction, are indicated in the legend. Curves between subjects were synchronized such that peak incremental shear strain occurred at 30% of the cardiac interval. The dotted black line represents the mean dADC over all subjects, whereas the gray shaded area indicates the SD. The largest dADC was observed in the feet–head direction
**Figure S3.** Correlation plots that show the relation between dADC and tissue strain, resulting from the combined GM‐WM tissue mask (Figures S4 and S5 show the correlation plots for dADC in WM and GM, respectively). The first and second rows show the relation of dADC with volumetric strain and shear strain, respectively. Coefficients of determination are indicated per subject in the legend. Measured dADC correlated best with shear strain. The coefficient of determination for the shear strain on the mean traces (solid lines, see Figure 5 in main text) was 0.85, 0.84, and 0.94 in the right‐to‐left (RL), anterior‐to‐posterior (AP), and feet‐to‐head (FH) direction, respectively. These coefficients were lower for the mean dADC versus volumetric strain: 0.43, 0.55 and 0.73, respectively
**Figure S4.** Correlation plots that show the relation between dADC and tissue strain for GM. The first and second rows show the relation of dADC with volumetric strain and shear strain, respectively. Coefficients of determination are indicated per subject in the legend. Measured dADC correlated best with shear strain. The coefficient of determination for the shear strain on the mean traces was 0.78, 0.55, and 0.93 in the RL, AP, and FH direction, respectively. These coefficients were lower for the mean dADC versus volumetric strain: 0.60, 0.47 and 0.87, respectively
**Figure S5.** Correlation plots that show the relation between dADC and tissue strain for WM. The first and second rows show the relation of dADC with volumetric strain and shear strain, respectively. Coefficients of determination are indicated per subject in the legend. Measured dADC correlated best with shear strain. The coefficient of determination for the shear strain on the mean traces was 0.86, 0.87, and 0.94 in the RL, AP, and FH direction, respectively. These coefficients were lower for the mean dADC versus volumetric strain: 0.33, 0.45 and 0.65, respectively
