# Supplementary material for: Dynamic brain ADC variations over the cardiac cycle and their relation to tissue strain assessed with DENSE at high‐field MRI
Source: Magn Reson Med. 2022 Mar 28;88(1):266–79. doi: 10.1002/mrm.29209 (PMC9315037; doi:10.1002/mrm.29209)
Supplement: Supplementary file 1 — Figure S1. Measured ADC deviation (dADC) curves over the cardiac cycle separately shown per acquisition orientation and encoding direction. Curves over the cardiac cycle were obtained by averaging over the conservative gray‐matter (GM) mask, avoiding blood and CSF signals. Mean ADC values over the cardiac cycle per subject, obtained for the associated acquisition orientation and encoding direction are indicated in the legend. Curves between subjects were synchronized such that peak incremental shear strain occurred at 30% of the cardiac interval. The dotted black line represents the mean dADC over all subjects, whereas the gray shaded area indicates the SD. The largest dADC was observed in the feet–head direction Figure S2. Measured dADC curves over the cardiac cycle separately shown per acquisition orientation and encoding direction. Curves over the cardiac cycle were obtained by averaging over the conservative white‐matter (WM) mask, avoiding blood and CSF signals. Mean ADC values over the cardiac cycle per subject, obtained for the associated acquisition orientation and encoding direction, are indicated in the legend. Curves between subjects were synchronized such that peak incremental shear strain occurred at 30% of the cardiac interval. The dotted black line represents the mean dADC over all subjects, whereas the gray shaded area indicates the SD. The largest dADC was observed in the feet–head direction Figure S3. Correlation plots that show the relation between dADC and tissue strain, resulting from the combined GM‐WM tissue mask (Figures S4 and S5 show the correlation plots for dADC in WM and GM, respectively). The first and second rows show the relation of dADC with volumetric strain and shear strain, respectively. Coefficients of determination are indicated per subject in the legend. Measured dADC correlated best with shear strain. The coefficient of determination for the shear strain on the mean traces (solid lines, see Figure 5 in main text) was 0.85, 0.8 [file MRM-88-266-s001.pdf]

Supporting Information

Infinitesimal strain

Over time, an elementary piece of brain tissue moves from position **X** at time $t = 0$ to position **x** at time $t = t'$. The coordinate **x** relates to **X** as follows: **x** = **X** + **u**(**X**,t), where **u** is the tissue displacement that includes rigid body rotations and translations. Hereafter t is omitted for brevity.

The *deformation gradient tensor* **F** relates a line element of tissue in the undeformed state to its deformed counterpart:

$$d\mathbf{x} = \mathbf{F}\, d\mathbf{X} \tag{S-1}$$

where **F** is given by

$$\mathbf{F} = \mathbf{I} + \nabla\mathbf{u} = \mathbf{I} + \mathbf{J} \tag{S-2}$$

Here, **I** is the identity matrix and **J** the *displacement gradient tensor*. The elements of **J** are the spatial derivatives of u_x, u_y and u_z; the measured incremental displacements in Right-to-Left (RL), Anterior-to-Posterior (AP) and Feet-to-Head (FH), respectively. Under the assumption of infinitesimal incremental strains, Cauchy's strain tensor can be derived from **F** as

$$\begin{aligned} \boldsymbol{\varepsilon} &= \frac{1}{2}\left(\mathbf{F}^{\mathrm{T}} + \mathbf{F}\right) - \mathbf{I} \\ &= \begin{pmatrix} \varepsilon_{xx} & \varepsilon_{xy} & \varepsilon_{xz} \\ \varepsilon_{yx} & \varepsilon_{yy} & \varepsilon_{yz} \\ \varepsilon_{zx} & \varepsilon_{zy} & \varepsilon_{zz} \end{pmatrix} \end{aligned} \tag{S-3}$$

where **ε** is symmetric and independent of rigid body translation or rotation. The assumption of infinitesimal incremental strain implies that the displacements of brain tissue are small, such that the geometry of the brain is macroscopically unchanged during the deformation process. As a result, any given voxel in the deformed state at position **x** can be assumed to coincide with its undeformed counterpart at position **X**, such that $\mathbf{x} = \mathbf{X} + \mathbf{u} \approx \mathbf{X}$. Using this assumption, the stretch tensor **U** can be expressed in terms of **ε**:

$$\mathbf{U} = \sqrt{\mathbf{F}^{\mathrm{T}}\mathbf{F}} = \sqrt{(\mathbf{I} + \mathbf{J})^{\mathrm{T}}(\mathbf{I} + \mathbf{J})} = \sqrt{\mathbf{I} + \mathbf{J} + \mathbf{J}^{\mathrm{T}} + \mathbf{J}^{\mathrm{T}} \cdot \mathbf{J}} \tag{S-4}$$

$$\approx \sqrt{\mathbf{I} + \mathbf{J} + \mathbf{J}^{\mathrm{T}}} \approx \mathbf{I} + \tfrac{1}{2}\left(\mathbf{J} + \mathbf{J}^{\mathrm{T}}\right) = \mathbf{I} + \boldsymbol{\varepsilon}$$

and similarly

$$\mathbf{U}^{-1} = \mathbf{I} - \boldsymbol{\varepsilon} \tag{S-5}$$

Linear approach for artificial ADC variation

We calculated the artificial deformation-induced magnitude variations of the MRI signal (see Eq. 6) for both b=300 (k_0=55mm^{-1}) and b=1000 (k_0=100mm^{-1}) in the associated direction. Since we measured ADC variations with DENSE in the RL, AP and FH direction, we only simulated the artificial ADC variations for these directions. Two components contribute to the variation: phase dispersion and the effective b-value. Calculating the phase dispersion effect is straight forward and can be done by expanding Eq. 9 to all three orthogonal directions (RL, AP and FH). The terms Δ_k that are required to express Eq. 8 in terms of the displacement gradient tensor $\mathbf{J}$ were obtained by substituting Eq. S-2 in Eq. 7

$$\mathbf{k} - \mathbf{k_0} = \left((\mathbf{I} + \mathbf{J})^{-1\mathrm{T}} - \mathbf{I}\right) \cdot \mathbf{k_0} \tag{S-6}$$

The effective b-value contribution, however, is more complex to derive and depends on the time-dependent inverse stretch $\mathbf{U}^{-1}(t)$. From the incremental displacement gradient tensor, we first computed the strain tensor following Eq. 3. By using Eq. 5, the stretch tensor $\mathbf{U}$ was obtained. In deriving the time-dependency of $\mathbf{U}$, we assumed a piece-wise constant strain rate over the evolution time Δ. From this assumption it follows that the incremental strain (that is, the strain at each time-point *t* over evolution time Δ) can be written as $\boldsymbol{\varepsilon}(t) = \frac{\boldsymbol{\varepsilon} \cdot t}{\Delta}$. The time-dependent inverse stretch tensor can then be written as

$$\mathbf{U}^{-1}(t) = \mathbf{I} - \boldsymbol{\varepsilon}(t) \tag{S-7}$$

With this, and by substituting Eq. S-5 into Eq. 11 we obtain (using Einstein summation convention)

$$\begin{aligned} D_{ij}^{obs} &= \left[\frac{1}{\Delta}\int_0^{\Delta}\left(I_{ik} - \frac{\varepsilon_{ik} \cdot t}{\Delta}\right) \cdot \left(I_{lj} - \frac{\varepsilon_{lj} \cdot t}{\Delta}\right) dt\right] D_{kl} \\ &= \left[I_{ik}\, I_{lj} - \frac{\varepsilon_{lj}}{2}\, I_{ik} - \frac{\varepsilon_{ik}}{2}\, I_{lj} + \frac{\varepsilon_{ik}\, \varepsilon_{lj}}{3}\right] D_{kl} \end{aligned} \tag{S-8}$$

Here, ε_{ik} and ε_{lj} is the measured strain after the evolution time Δ, which varies for each cardiac phase. For a given, constant **D** (measured through the ungated DTI) we obtain a prediction for $\mathbf{D}^{\mathbf{obs}}$ that varies over the cardiac cycle.

Strain analysis

The reconstruction of the gradient displacement tensor $\mathbf{J}$ was previously reported [3]. For completeness, we summarize the processing steps as follows. Each DENSE series provided two components of the gradient displacement tensor $\mathbf{J}$. For instance, the sagittal oriented dataset with FH encoding direction from Figure 2 in the main text provided the incremental displacement gradient fields $\frac{\partial u_{FH}}{\partial FH}$ and $\frac{\partial u_{FH}}{\partial AP}$. The diagonal elements of the displacement gradient tensor were obtained twice, each for a different acquisition orientation. Each double obtained element was averaged to obtain the full gradient displacement tensor, from which the deformation gradient tensor **F** (Eq. S-2) could be derived. Substituting **F** in Eq. S-3 yielded Cauchy's strain tensor. From Cauchy's strain tensor, we derived two scalar quantities for comparison with the ADC variations: the incremental volumetric strain and the incremental octahedral shear strain [3]. The volumetric strain reflects the net incremental expansion or compression of the voxel [1]. Octahedral shear strain, on the other hand, signifies the differences between the three principal strains, which can be interpreted as the deviation from isotropic swelling or shrinkage [2].

Supporting Figures

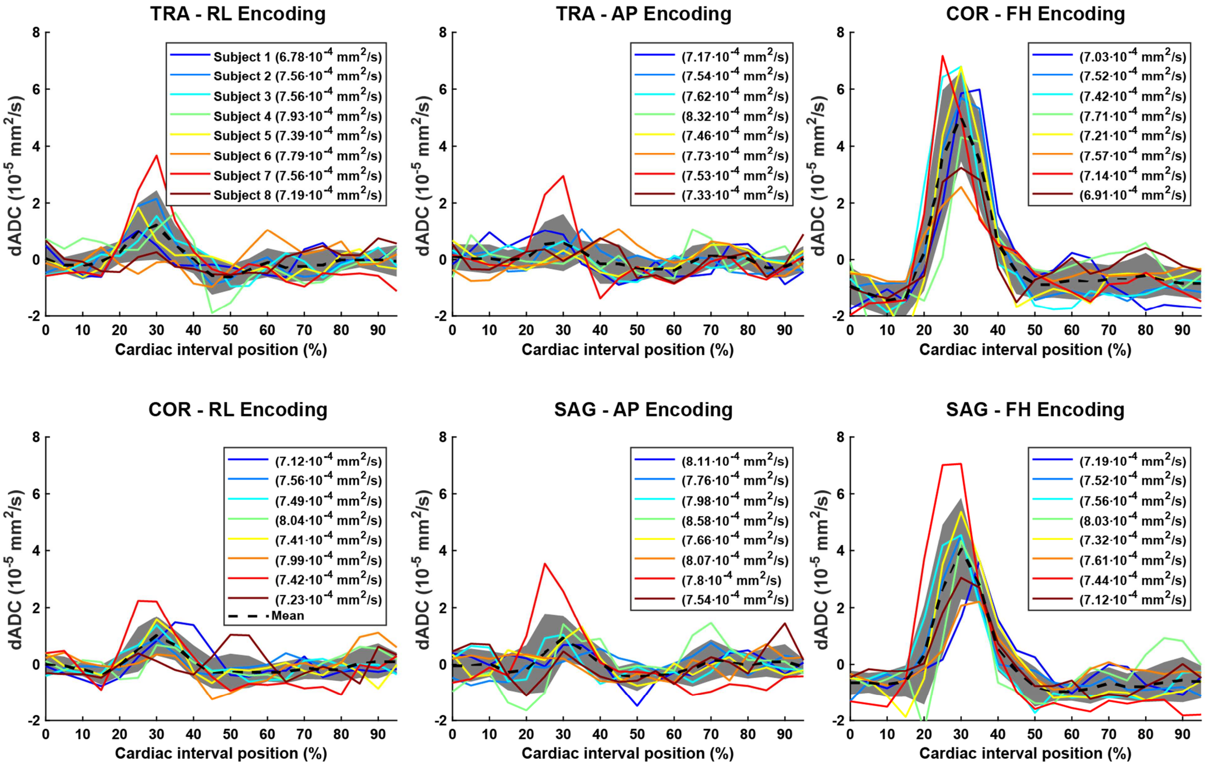

Figure S1 *Measured dADC curves over the cardiac cycle separately shown per acquisition orientation and encoding direction. Curves over the cardiac cycle were obtained by averaging over the conservative grey matter (GM) mask, avoiding blood and CSF signals. Mean ADC values over the cardiac cycle per subject, obtained for the associated acquisition orientation and encoding direction are indicated in the legend. Curves between subjects were synchronized such that peak incremental shear strain occurred at 30% of the cardiac interval. The dotted black line represents the mean dADC over all subjects whereas the gray shaded area indicates the standard deviation. The largest dADC was observed in the Feet-to-Head direction.*

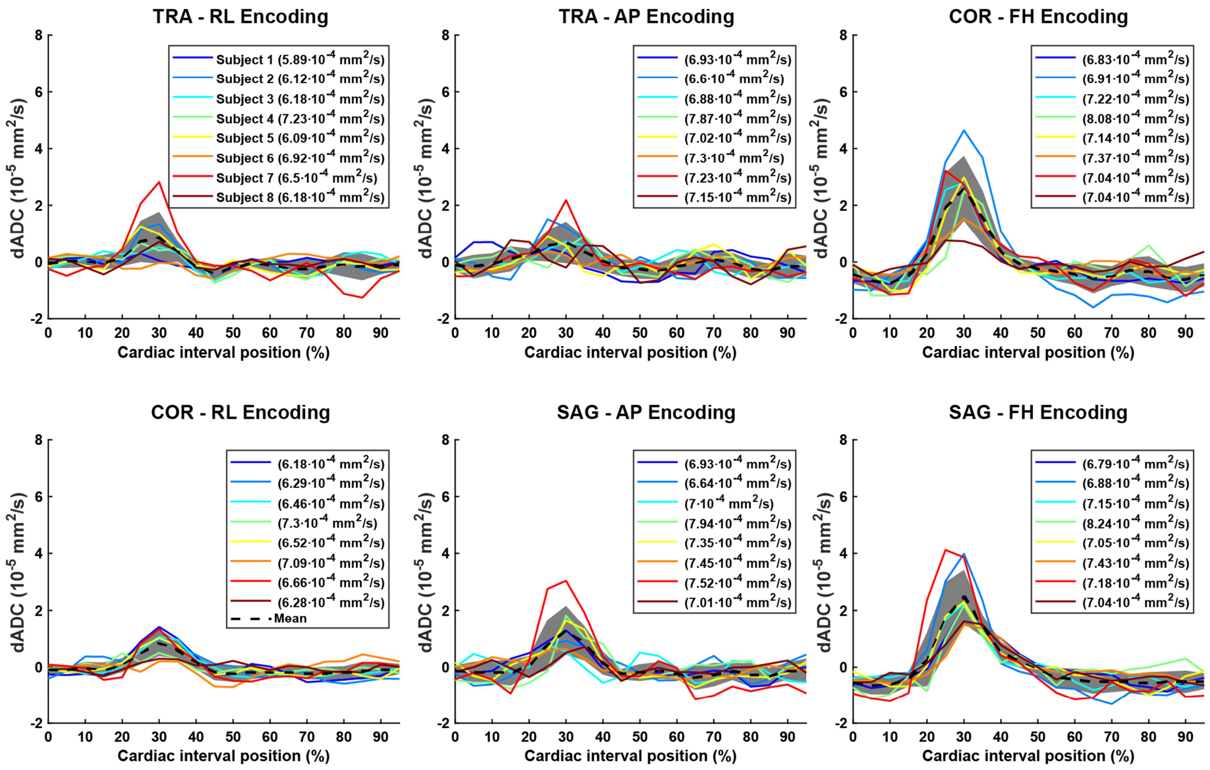

Figure S2 *Measured dADC curves over the cardiac cycle separately shown per acquisition orientation and encoding direction. Curves over the cardiac cycle were obtained by averaging over the conservative white matter (WM) mask, avoiding blood and CSF signals. Mean ADC values over the cardiac cycle per subject, obtained for the associated acquisition orientation and encoding direction are indicated in the legend. Curves between subjects were synchronized such that peak incremental shear strain occurred at 30% of the cardiac interval. The dotted black line represents the mean dADC over all subjects whereas the gray shaded area indicates the standard deviation. The largest dADC was observed in the Feet-to-Head direction.*

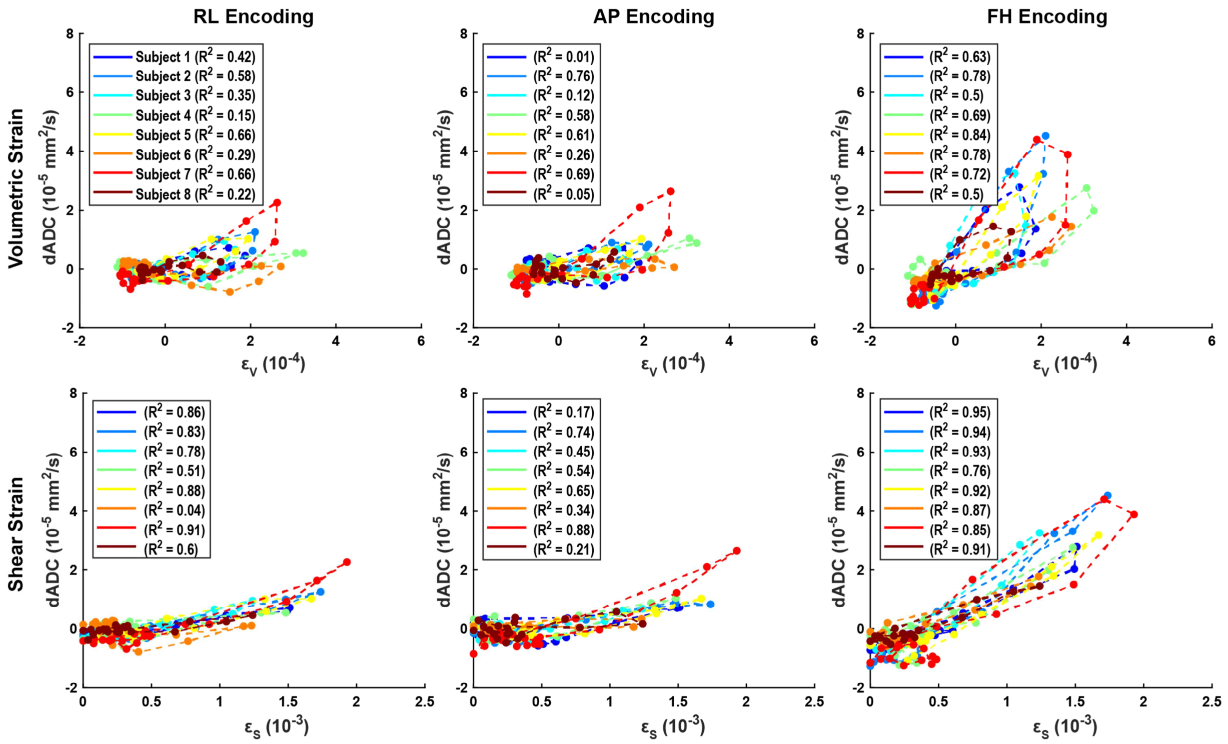

Figure S3 *Correlation plots that show the relation between dADC and tissue strain, resulting from the combined GM-WM tissue mask (Figure S4 and S5 show the correlation plots for dADC in WM and GM, respectively). The first and second rows show the relation of dADC with volumetric strain and shear strain, respectively. Coefficients of determination are indicated per subject in the legend. Measured dADC correlated best with shear strain. The coefficient of determination for the shear strain on the mean traces (solid lines, see Figure 5 in main text) was 0.85, 0.84 and 0.94 in the RL, AP and FH direction, respectively. These coefficients were lower for the mean dADC versus volumetric strain: 0.43, 0.55 and 0.73, respectively.*

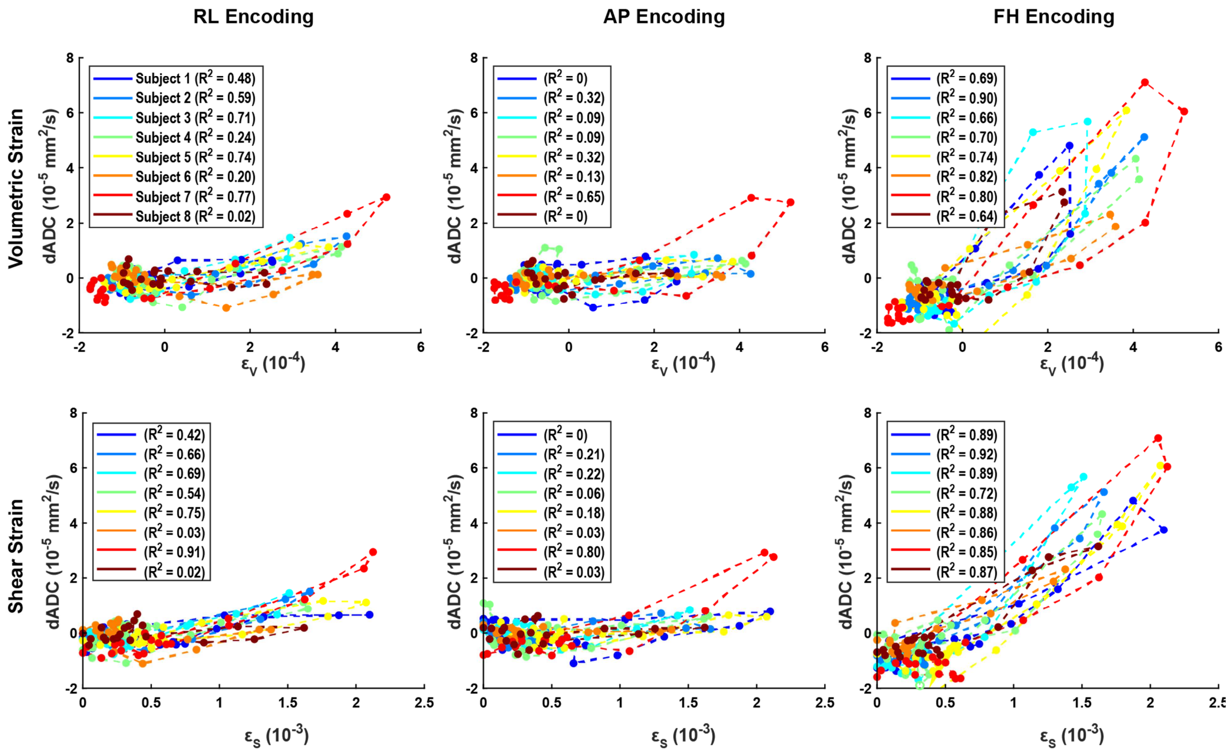

Figure S4 *Correlation plots that show the relation between dADC and tissue strain for GM. The first and second rows show the relation of dADC with volumetric strain and shear strain, respectively. Coefficients of determination are indicated per subject in the legend. Measured dADC correlated best with shear strain. The coefficient of determination for the shear strain on the mean traces was 0.78, 0.55 and 0.93 in the RL, AP and FH direction, respectively. These coefficients were lower for the mean dADC versus volumetric strain: 0.60, 0.47 and 0.87, respectively.*

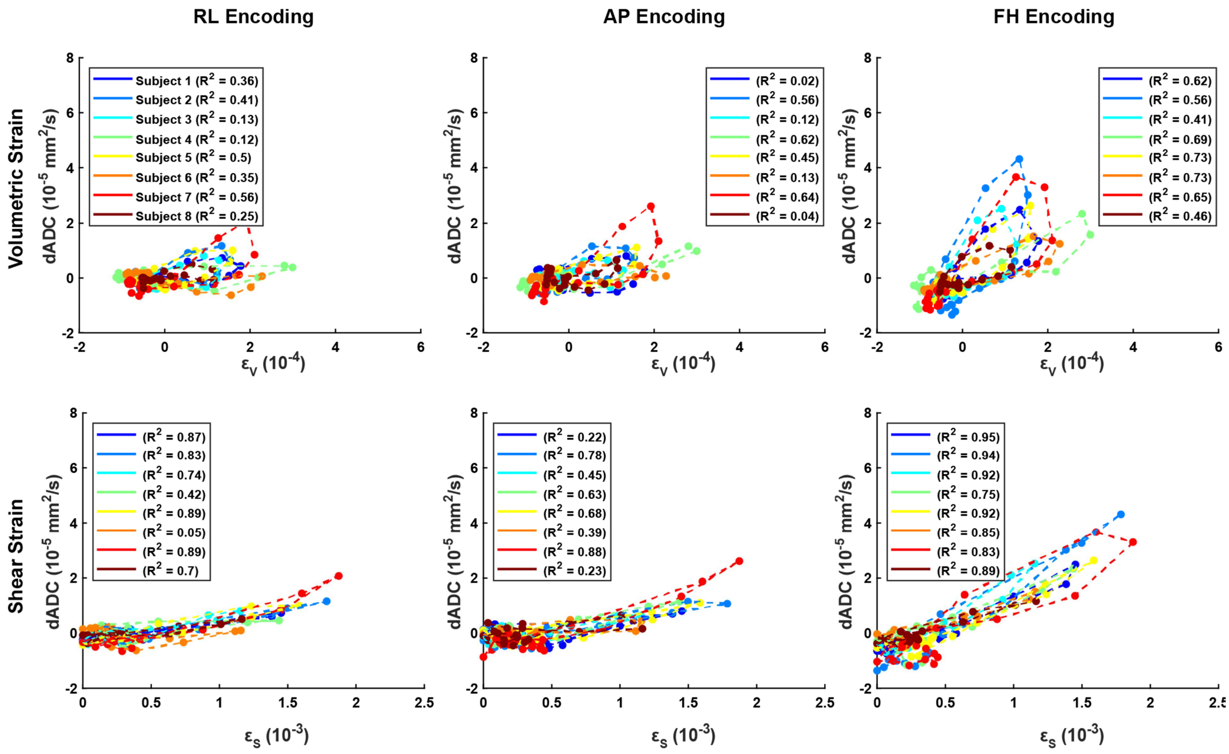

Figure S5 *Correlation plots that show the relation between dADC and tissue strain for WM. The first and second rows show the relation of dADC with volumetric strain and shear strain, respectively. Coefficients of determination are indicated per subject in the legend. Measured dADC correlated best with shear strain. The coefficient of determination for the shear strain on the mean traces was 0.86, 0.87 and 0.94 in the RL, AP and FH direction, respectively. These coefficients were lower for the mean dADC versus volumetric strain: 0.33, 0.45 and 0.65, respectively.*

References

1. **Adams, A. L., Kuijf, H. J., Viergever, M. A., Luijten, P. R. & Zwanenburg, J. J. M.** Quantifying cardiac-induced brain tissue expansion using DENSE. *NMR Biomed.* 32: e4050 (2019).
2. **McGarry, M. D. J. J. *et al.*** An octahedral shear strain-based measure of SNR for 3D MR elastography. *Phys. Med. Biol.* 56: N153-64 (2011).
3. **Sloots, J. J., Biessels, G. J., de Luca, A. & Zwanenburg, J. J. M.** Strain Tensor Imaging: Cardiac-induced brain tissue deformation in humans quantified with high-field MRI. *Neuroimage* 236: 118078 (2021).
